# Metagenome of the Microbial Community of Anammox Granules in a Nitritation/Anammox Wastewater Treatment System

**DOI:** 10.1128/genomeA.01115-17

**Published:** 2017-10-19

**Authors:** Andrey V. Mardanov, Alexey V. Beletsky, Yury Nikolaev, Roman Y. Kotlyarov, Anna Kallistova, Nikolai V. Pimenov, Nikolai V. Ravin

**Affiliations:** a Institute of Bioengineering, Research Center of Biotechnology of the Russian Academy of Sciences, Moscow, Russia; b Winogradsky Institute of Microbiology, Research Center of Biotechnology of the Russian Academy of Sciences, Moscow, Russia; c Wastewater and Sludge Treatment Division of Engineering and Technology Center, JSC Mosvodokanal, Moscow, Russia

## Abstract

We sequenced the metagenome of a granular sludge in a nitritation/anammox bioreactor used for the treatment of ammonium-rich wastewater. *Proteobacteria*, *Planctomycetes*, *Bacteroidetes*, *Chloroflexi*, *Ignavibacteriae*, and *Acidobacteria* were the predominant phyla in the studied bioreactor. Binning of contigs yielded a near-complete genome of the dominant anammox bacterium assigned to the candidate genus *Brocadia*.

## GENOME ANNOUNCEMENT

Partial nitritation/anammox (N/A) technologies are promising for the treatment of ammonium-rich wastewater. However, the anammox bacteria are characterized by very low growth rates, which limits the scale-up of the anammox process, while the absence of pure cultures of these bacteria complicates an investigation of their genetic and physiological characteristics. Moreover, anammox bacteria are highly sensitive to a number of environmental factors (temperature, pH, etc.) that cause instability of the anammox process (1). The implementation of N/A technologies is also impeded by the complexity of microbial communities of bioreactors comprising not only anammox bacteria, but also a number of other physiological groups, such as fermentative bacteria, nitrifiers, denitrifiers, methanogens, and others (2–4).

In this study, a 100-liter hybrid completely mixed constant-flow N/A reactor with suspended and immobilized activated sludge operating at a Moscow wastewater treatment plant (5) was analyzed. The 50-g sample of firmly attached sludge, representing anammox granules, was isolated on day 120 of the reactor operation when it stably removed about 81% of the total nitrogen with an influent ammonium concentration of 270 to 300 mg NH_4_-N/liter (5). Metagenomic DNA was isolated using the PowerSoil DNA isolation kit (Mo Bio, Inc. Laboratories, Carlsbad, CA) and sequenced with a Roche Genome Sequencer FLX (GS FLX) using the Titanium XL+ protocol for a shotgun library and paired-end library, with an average size of 8 kbp. About 312 Mb and 61 Mb of sequences were obtained for the shotgun and the paired-end libraries, respectively. To characterize the taxonomic composition of the microbial community, we identified raw pyrosequencing reads representing 16S rRNA genes using a BLASTN search against the SILVA SEED database (6). The RDP Classifier (7) was used for the taxonomic classification of these sequences. *Proteobacteria*, mostly represented by the class *Betaproteobacteria*, was the most abundant lineage (26.8% of all reads classified at the phylum level), followed by *Planctomycetes* (20.0%), *Bacteroidetes* (14%), *Chloroflexi* (11%), *Ignavibacteriae* (6%), and *Acidobacteria* (5%). The presence of these bacterial lineages reflects the complexity of microbial processes in the studied N/A bioreactor performed by anammox *Planctomycetes*, fermentative bacteria, and denitrifiers. Archaea accounted for only 1% of 16S rRNA reads and were represented by methanogens of the order *Methanobacteriales*.

The reads were *de novo* assembled into contigs using the Newbler Assembler version 2.9 (454 Life Sciences, Branford, CT). A total of 7,798 contigs with an *N*_50_ contig size of 2,137 bp were obtained. Binning of contigs using the CONCOCT tool (8) allowed us to obtain a near-complete composite genome of the dominant anammox bacterium. A BLASTN search against the NCBI database revealed that 16S rRNA sequence from this genome is 97% identical to that of *Candidatus* “Brocadia caroliniensis” (2). The obtained genomic data will be useful for further studies of microbial processes in wastewater treatment systems and genome-based analysis of anammox bacteria.

### Accession number(s).

The sequences obtained in this project have been deposited in the NCBI Sequence Read Archive under the accession numbers SRX3153926, SRX3153925, and SRX3153924.
